# Influence of a genetic variant of *CHAT* gene over the profile of plasma soluble ChAT in Alzheimer disease

**DOI:** 10.1590/1678-4685-GMB-2019-0404

**Published:** 2020-11-20

**Authors:** Patricia Fernanda Rocha-Dias, Daiane Priscila Simao-Silva, Saritha Suellen Lopes da Silva, Mauro Roberto Piovezan, Ricardo Krause M. Souza, Taher. Darreh-Shori, Lupe Furtado-Alle, Ricardo Lehtonen Rodrigues Souza

**Affiliations:** 1 Universidade Federal do Paraná (UFPR), Centro Politécnico, Programa de Pós-Graduação em Genética, Departamento de Genética, Curitiba, PR, Brazil.; 2 Instituto de Pesquisa do Câncer (IPEC), Guarapuava, PR, Brazil.; 3 Universidade Federal do Paraná (UFPR), Departamento de Neurologia, Hospital de Clínicas, Curitiba, PR, Brazil.; 4 Instituto de Neurologia de Curitiba (INC), Ambulatório de Distúrbios da Memória e Comportamento, Demência e Outros Transtornos Cognitivos e Comportamentais, Curitiba, PR, Brazil.; 5 Karolinska Institutet, Care Sciences and Society, Department of Neurobiology, Stockholm, Sweden.

**Keywords:** ChAT, VAChT, dementia, cholinergic dysfunction, peripheral cholinergic signaling

## Abstract

The choline acetyltransferase (ChAT) and vesicular acetylcholine transporter (VAChT) are fundamental to neurophysiological functions of the central cholinergic system. We confirmed and quantified the presence of extracellular ChAT protein in human plasma and also characterized ChAT and VAChT polymorphisms, protein and activity levels in plasma of Alzheimer's disease patients (AD; N = 112) and in cognitively healthy controls (EC; N = 118). We found no significant differences in plasma levels of ChAT activity and protein between AD and EC groups. Although no differences were observed in plasma ChAT activity and protein concentration among ChEI-treated and untreated AD patients, ChAT activity and protein levels variance in plasma were higher among the rivastigmine-treated group (ChAT protein: p = 0.005; ChAT activity: p = 0.0002). Moreover, AD patients homozygous for SNP rs1880676 *A* allele exhibited higher levels of ChAT activity. Considering this is the first study to report the influence of genetic variability of CHAT locus over ChAT activity in AD patients plasma, it opens a new set of important questions on peripheral cholinergic signaling in AD.

The central cholinergic system plays a fundamental role in memory and learning mechanisms, and cholinergic deficit in Alzheimer’s disease (AD) generates cognitive impairment (Bartus *et al.,* 1982). The “cholinergic hypothesis” (Davies and Maloney, 1976) refer to the cholinergic deficits in AD and the inability to transmit neurologic impulses across brain synapses. The cholinesterases (ChE) are a family of enzymes that catalyze the hydrolysis of acetylcholine (ACh) into choline and acetic acid, an essential process for the restoration of the cholinergic neuron. There are two cholinesterase types: acetylcholinesterase (AChE; EC 3.1.1.7) and butyrylcholinesterase (BChE; EC 3.1.1.8). Both enzymes participate in cholinergic neutrotransmission by hydrolyzing acetylcholine in the central and peripheral nervous systems (Pohanka, 2011). Based on deficits in AD, cholinesterase inhibitors (ChEIs) are the first-line drugs in the symptomatic treatment of AD by inhibiting cholinesterase, and thus resulting in increased synaptic levels of ACh neurotransmitter. Currently the most prescribed ChEIs are donepezil, galantamine and rivastigmine (Ferris *et al.,* 2013).

Cholinergic dysfunction is characterized by severe reduction in the cholinergic enzyme choline acetyltransferase (ChAT), which is one of the key features of the brains of patients with AD (Boissière *et al.,* 1997). ChAT is responsible for the biosynthesis of the cholinergic neurotransmitter and acetylcholine (ACh) (Lee *et al.*, 2012). ChAT reversibly catalyzes the transfer of the acetyl group from acetyl-coenzyme A to a choline molecule. Subsequently, the cytoplasmic ACh is stored in synaptic vesicles by the vesicular acetylcholine transporter (VAChT), until its release into the synaptic cleft (Oda, 1999; Govindasamy *et al.,* 2004).

ChAT and VAChT have an important role in neurophysiological functions, especially for a correct performance of the cholinergic system (Nordberg and Svensson, 1998), being encoded by the genes *CHAT* and *SLC18A3*, respectively. The central cholinergic system influences a wide range of neurophysiological processes, including cognitive performance, arousal, sleep, movement and processing of visual information (Oda, 1999).

Wilcock *et al.* (1982) found a decrease in ChAT activity, in the temporal and frontal lobe in the AD brain (Wilcock *et al.,* 1982). In patients with mild cognitive impairment (MCI), however, an upregulation of ChAT activity is reported in the hippocampus and frontal cortex (DeKosky *et al.,* 2002).

*CHAT* gene and protein expression are reduced in AD when compared with the controls group brain (González-Castañeda *et al.,* 2013). Low ChAT protein levels have been shown to correlate with the severity of AD assessed by neuropsychological measures (Baskin *et al.*, 1999), as well as severity of neuropathological lesions (Davis *et al.,* 1999).

ChAT is considered a cytosolic enzyme found in both neurons (Bellier and Kimura, 2011; Lee et al. 2012), and in several non-neuronal cells (Hersh and Peet, 1978; Sastry *et al.,* 1981; Kawashima and Fujii, 2003). Recent studies provide evidences for the presence of ChAT activity and protein in extracellular fluids, such as human plasma and cerebrospinal fluid (Vijayaraghavan *et al.,* 2013), reformulating the concept that ChAT acts only as a cytosolic enzyme, located in cholinergic terminals (Oda,1999; Govindasamy *et al.,* 2004).

In this study both, activity and the protein concentration of ChAT were measured in plasma samples of a patients with AD and control group of a Brazilian cohort, composed by 230 plasma samples of Brazilian individuals (112 cases with clinical diagnoses of AD and 118 cognitively normal elderly controls (EC), predominantly euro descendants). The samples were from the Cognitive Dysfunction Ambulatory from Clinical Hospital of the Federal University of Parana (HC-UFPR) and Disorders Clinic Memory and Behavior (ADEMEC) of Curitiba Neurology Institute (INC).

The diagnostic criteria for patients with AD followed the NINCDS-ADRDA standards (National Institute of Neurological and Communicative Disorders and Stroke-Alzheimer's disease and Related Disorders Association; McKhann *et al.* (2011), with adaptations of the recommendations of the Scientific Department of Neurology cognitive and aging of the Brazilian Academy of Neurology (Frota *et al.,* 2011). Exclusion criteria for AD were: other forms of dementia, other psychiatric disorders, changes in recommended subsidiary exams, or any evidence or suspicion of inflammatory or infectious CNS disease. 

Patients of the control group were excluded if they had infectious diseases (hepatitis, malaria, Chagas disease, HIV), were alcoholics, had previous history of stroke or loss of memory lapse, unable to perform daily life activities, had persistent complaints about memory and depressive symptoms.

In the current study, the global cognition was assessed by the Mini-Mental State Examination tests, MMSE (Folstein *et al.,* 1975), while the stage of the disease was determined by the Clinical Dementia Rating scale, CDR (Morris, 1993). The patients with AD, and/or their kin or legal guardians, as well as the patients of the control group, were informed about the research, and if agreed, signed the Term of Free-informed to participate in the study. The project was approved by Ethics Committee of the Federal University of Parana, Health Sciences Sector.

We also determined the genetic polymorphism in the cholinergic locus (the *rs3810950, rs733722,* and *rs1880676* for *CHAT*) and *rs2269338* for the *SLC18A3* gene (VAChT). Then we investigated whether these genetic variants influence ChAT profiles in the plasma samples of this Brazilian cohort, in relation to education, cognitive performance and the use of cholinesterase inhibitors (ChEIs).

Genotyping was performed by TaqMan SNP Genotyping Assays (Applied Biosystems) in a ViiA 7 Real-Time PCR System (Thermofisher Scientific). For this, the total genomic DNA was extracted from peripheral blood by a salting out method (Lahiri and Nurnberger Jr, 1991) (with modifications) and diluted to a final concentration of 20 ng/μL. The PCR conditions used for each individual SNP were composed of 5 μL TaqMan Universal PCR Master Mix, 0.5 µL of the specific SNP probes included in each TaqMan Kit, 2.5 µL of ultrapure water and 2 µL DNA 20 ng/µL, having a final volume of 10 μL. The PCR cycles were 60 s at 60 °C; 10 min at 95 °C; 50 times 15 cycles at 95 °C alternated with 90 s at 60 °C; and a final step of 30 sat 60°C.

To determine the levels of ChAT in plasma we performed an integrated enzyme activity-sandwich ELISA-(Enzyme Linked Immunosorbent Assay) set up as described previously (Vijayaraghavan *et al*., 2013). These analyses were done at Karolinska Instituted, the Department of Neurobiology, Care Sciences and Society, Stockholm, Sweden.

Nortest package R program was used to test for normality (Shapiro-Wilk test with Lilliefors correction) of the variables activity and protein concentration, MMSE and years of study. After analyzing the distributions, comparisons were made by means of the *t* test or Mann-Whitney for parametric and nonparametric variables, respectively, as well as multiple regression analysis. Variances were compared by the Bartlett test. The confidence interval for all statistical analyzes was 95% (p = 0.05).

The demographic data of the AD patients and the EC are presented in Table 1. The mean age was significantly different between patients with AD and EC. This is due to the difficulty related to finding cognitively healthy volunteers following the exclusion criteria described in the method section. The MMSE data corroborate an expected difference between AD and EC groups. The EC group had more years of education, most likely reflecting the younger pattern of age among the EC group. Approximately 90% (n = 101) of the AD patients underwent pharmacological treatment for dementia, and about 70% (n = 71) of the AD medication was ChEIs.

**Table 1 -:** Demographics and characteristics of Alzheimer patients (AD) and elderly controls (EC).

	AD	EC	
	N = 112	N = 118	
	Median	Median	p
	(1st quartile - 3rd quartile)	(1st quartile - 3rd quartile)	
Age	79 (73 - 84)	71 (66 - 76)	5.8e-8
MMSE	15 (6.75 - 20)	27 (26 - 29)	2.2e-16
Education*	4 (2 - 8)	4 (4 - 11)	0.027
Female (F)	60.7%	72.9%	0.049
Male (M)	39.3%	27.1%	
AD Treatment	89.3%	- - -	
ChEIs	69.6%	- - -	

* years of study.

The median together with 1^st^ and 3^rd^ quartiles values of the protein concentration and activity of ChAT in the plasma samples of AD patients and EC is presented in Table 2. No significant difference between the groups was observed.

**Table 2 -:** Protein and activity of soluble choline acetyltransferase (ChAT) in plasma of patients with AD and elderly controls (EC).

	AD N=112	EC N=118	AD x EC
	Median	Median	p
	(1st quartile - 3rd quartile)	(1st quartile - 3rd quartile)	
Plasma ChAT protein (μg/mL)	96.74 (55.07 - 139.94)	85.73 (58.17 -118.64)	0.46
Plasma ChAT activity (nmol/min/mL)	33.29 (20.02 - 46.92)	28.82 (16.21 - 43.63)	0.11

Unlike the ChAT levels in the brain of patients with AD, the plasma pattern of ChAT is not known, so this is the first report on ChAT levels and how they differed in the brain (Boissière *et al.,* 1997). The relation between peripheral ChAT and AD is established by the classical neurotransmitter ACh. This molecule acts as a suppressor of inflammatory responses of lymphocytes (Parrish *et al.,* 2008), and the major sources of extracellular ChAT in the plasma might be lymphocytes (Vijayaraghavan *et al.,* 2013). The systemic immunity is modulated by the cholinergic anti-inflammatory pathway (Pavlov *et al.,* 2009) and the cholinergic transmission in turn cause low-degree systemic inflammation in DA (Darreh-Shori, *et al.* 2009). Therefore, a hypothesis would be to alter the expression levels of ChAT, just as it occurs in the brain. In this study for the patients with AD, the peripheral level of ChAT concentration may have been influenced by the action of IChEs, since the enzyme is modulated by the bioavailable Ach concentration. Possibly the changes caused in the inflammatory pathway do not change the peripheral ChAT synthesis.

These data were analyzed with respect to ChAT gene polymorphism profiles, presented in Table 3. Among the AD group, a significant difference was observed in the plasma ChAT activity with respect to the SNP, *rs1880676.* The AD carriers of the G allele had significantly lower plasma ChAT activity than the non-carrier AD group (p = 0.002, Table 3).

**Table 3 -:** Comparisons between the median of ChAT protein concentration and activity in plasma, grouped by carrier and not allele carrier, for each specific SNP (*rs3810950*, *rs2269338*, *rs1880676* and *rs733722*) from (ADxAD; ECxEC).

*rs3810950*	AD			EC		
	Carrier G (AG+GG)	Not Carrier (AA)	p	Carrier G (AG+GG)	Not Carrier (AA)	p
	N=99	N= 5		N= 81	N= 6	
ChAT protein	91.70	148.87	0.23	81.53	54.24	0.10
ChAT activity	33.29	39.78	0.52	28.24\	19.83	0.48
*rs1880676*	AD			EC		
	Carrier G (AG+GG)	Not Carrier (AA)	p	Carrier G (AG+GG)	Not Carrier (AA)	p
	N= 98	N= 5		N= 78	N= 7	
ChAT protein	91.53	148.87	0.24	80.97	47.52	0.05
ChAT activity	33.58	39.78	0.002	27.68	28.36	0.78
*rs733722*	AD			EC		
	Carrier G (GT+GG)	Not Carrier (TT)	p	Carrier G (GT+GG)	Not Carrier (TT)	p
	N= 93	N= 9		N= 84	N= 3	
ChAT protein	96.28	90.85	0.42	77.47	101.36	0.96
ChAT activity	33.59	30.22	0.84	28.32	31.91	0.32
rs2269338*	AD			EC		
	Carrier G (GT+GG)	Not Carrier (TT)	p	Carrier G (GT+GG)	Not Carrier (TT)	p
	N= 102	N= 3		N= 86	N= 2	
ChAT protein	91.70	206.24	0.08	77.47	224.10	0.09
ChAT activity	32.51	45.33	0.12	28.32	5.85	0.12

ChAT protein concentration is in ng/mL and the enzyme activity in nmol/min/ml. The SNP *rs3810948* was not included because it presents almost all the samples only one genotype. *The SNP, *rs2269338* is in the *SLC18A3* gene, commonly known as VACHT gene (vesicular acetylcholine transporter).

No differences were observed in ChAT activity (p = 0.67) and ChAT protein concentration in plasma (p = 0.79) between patients with and without treatment with CHEIs. However, separating by type of treatment (Figure 1), although means do not differ, the variance of the group that used rivastigmine is higher (ChAT protein: p = 0.005; ChAT activity: p = 0.0002).

**Figure 1 -:**
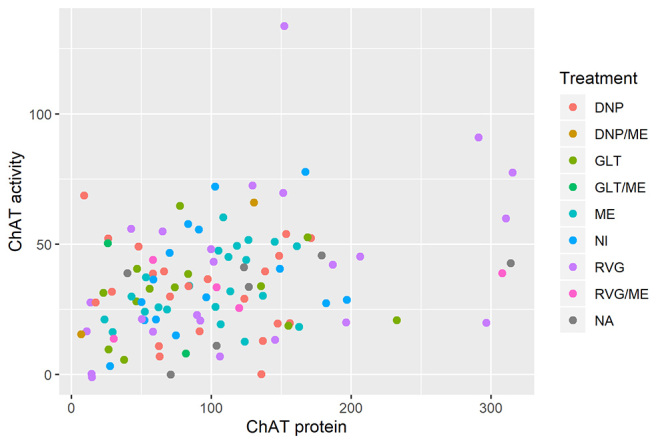
Comparisons of the ChAT protein and activity of soluble ChAT in plasma of patients with Alzheimer’s disease (AD), according to the treatment type. NA: no information; NI: do not use medicine; ME: memantine; DNP: donepezil; GLT: galantamine; RVG: rivastigmine.

About 70% of the AD patients, included in the current study, were on treatment with the ChEIs, which are the main therapeutic option available today. Currently, there is no information on how treatment with ChEIs may affect the levels of soluble ChAT in plasma, cerebrospinal fluid or the brain. Therefore, the lack of difference between the plasma ChAT levels among the AD and the controls could be due to an increase in ChAT levels in plasma, possibly induced by treatment with ChEIs. This is because ChEIs are expected to alter ACh homeostasis by inhibiting degradation of ACh by cholinesterases in circulation (Vijayaraghavan *et al.,* 2013). The plasma ChAT levels between the AD patients who were on ChEIs therapy, compared to those who were not, partially supported the above notion, since the variance of ChAT was different in those taking rivastigmine, which indicates a bidirectional influence of this drug on ChAT protein expression in the plasma. This is interesting since BChE activity dominates in plasma, and rivastigmine is the only ChEI as AD therapeutic that inhibits both AChE and BChE with equal efficacy (Darreh-Shori *et al.,* 2002). In whole-blood circulation, AChE activity dominates as attached on the outer cell membrane of the red blood cells (RBC), and BChE as soluble enzyme in plasma fluid. In case of donepezil and galantamine treatments, mainly RBC AChE activity is inhibited by about 30-40% (Darreh-Shori *et al.,* 2006 *,* 2008). Thus the unaffected plasma BChE is more than enough to compensate since these two ChEIs have negligible activity on plasma BChE (Darreh-Shori *et al.,* 2006, 2008). In contrast, rivastigmine inhibits both AChE and BChE with over 40% at the recommended dosage (Darreh-Shori *et al.,* 2002). In addition the efficacy of inhibition by rivastigmine can greatly vary in different patients depending on variables such as tolerated dose and body weight, which in turn result in a large variation in the inhibition levels of these two enzymes. This could express itself in a wide variation in the plasma ChAT expression as was seen among the rivastigmine-treated patients. More studies are however required to confirm and expand the current findings.

In conclusion, we reaffirm the presence of ChAT in human plasma by measuring both protein and activity of this enzyme in a reasonably large number of samples from patients with AD and the control group. Albeit no difference between plasma levels of ChAT among the groups were observed, we found that the *rs1880676* SNP alters differentially the phenotypic profiles of ChAT activity and protein expression in the plasma of the AD patients. This is the first study to report the influence of genetic variant of the cholinergic locus with the profile of soluble ChAT in plasma. Overall, the findings warrant further studies since identification and understanding of factors that may influence the phenotypic profile of ChAT in plasma could be important for the understanding of the role of this soluble enzyme in the normal and pathological function of peripheral cholinergic signaling.
